# Characterization of a molecular switch system that regulates gene expression in mammalian cells through a small molecule

**DOI:** 10.1186/1472-6750-10-15

**Published:** 2010-02-18

**Authors:** Jennifer L Taylor, Priyanka Rohatgi, H Trent Spencer, Donald F Doyle, Bahareh Azizi

**Affiliations:** 1 School of Chemistry and Biochemistry, Georgia Institute of Technology, 901 Atlantic Drive, Atlanta, GA 30332, USA; 2 Pharmacokinetics and ADME group, Baxter Healthcare, One Baxter Parkway Deerfield, IL 60015-4625, USA; 3 Aflac Cancer Center and Blood Disorders Services, Department of Pediatrics, Emory University School of Medicine, Atlanta, GA 30322, USA

## Abstract

**Background:**

Molecular switch systems that activate gene expression by a small molecule are effective technologies that are widely used in applied biological research. Nuclear receptors are valuable candidates for these regulation systems due to their functional role as ligand activated transcription factors. Previously, our group engineered a variant of the retinoid × receptor to be responsive to the synthetic compound, LG335, but not responsive to its natural ligand, 9-*cis*-retinoic acid.

**Results:**

This work focuses on characterizing a molecular switch system that quantitatively controls transgene expression. This system is composed of an orthogonal ligand/nuclear receptor pair, LG335 and GRQCIMFI, along with an artificial promoter controlling expression of a target transgene. GRQCIMFI is composed of the fusion of the DNA binding domain of the yeast transcription factor, Gal4, and a retinoid × receptor variant. The variant consists of the following mutations: Q275C, I310M, and F313I in the ligand binding domain. When introduced into mammalian cell culture, the switch shows luciferase activity at concentrations as low as 100 nM of LG335 with a 6.3 ± 1.7-fold induction ratio. The developed one-component system activates transgene expression when introduced transiently or virally.

**Conclusions:**

We have successfully shown that this system can induce tightly controlled transgene expression and can be used for transient transfections or retroviral transductions in mammalian cell culture. Further characterization is needed for gene therapy applications.

## Background

Gene regulation systems are important research tools for studying gene function and can provide numerous benefits for clinical applications. Several systems have been designed that place a target transgene under the control of an engineered transcription factor that is activated in the presence of an exogenous ligand [1]. These systems have been successfully used to control expression of a target transgene in a cellular environment with high expression levels in response to an extensive range of ligand concentrations [2]. To date, several research groups have used these systems to control transgene expression in both cell culture as well as animal models. Some of the most commonly used examples include the progesterone receptor (PR)/mifepristone (RU486) inducible system [3], the tetracycline inducible system [4], and the ecdysone-responsive regulation system [5].

The progesterone receptor (PR) inducible system, also known as GeneSwitch^®^, regulates gene expression using low concentrations of RU486 that binds to a chimeric regulator composed of a truncated PR fused to a Gal4 DNA binding domain and p65, the activation domain [3,6-8]. Upon the addition of RU486, the regulator then binds a DNA sequence composed of six Gal4 response elements (RE) and activates gene expression. One disadvantage of this system is that the ligand, RU486, has been shown to interfere with other biological pathways (at a much higher concentration of ligand), so long term usage could have extensive side effects [9]. In the tetracycline (Tet) inducible system, the prokaryotic protein, Tet, binds to a specific DNA sequence called *tetO *in response to tetracycline or doxycycline (dox) [4]. This system can function as both an ON-switch as well as an OFF-switch [10]. The ligand dox is inexpensive and "bioavailable" [11]. Since this system utilizes bacterial proteins, an immunogenic response may occur if used in human gene therapy [12,13]. Finally, the ecdysone-responsive regulation system (also known as RheoSwitch^®^) is based on a heterodimer between the insect steroid hormone receptor, ecdysone receptor (EcR), and the retinoid × receptor (RXR) [14]. Despite low basal expression and high fold induction [15,16], this system requires the over expression of two transgenes (EcR and RXR) simultaneously, complicating viral delivery or a single component system. Another disadvantage would be that over expression of RXR poses a safety concern as RXR is involved in many metabolic pathways [17]. The concerns posed by these systems permit the development of new or improved molecular switch systems.

According to Toniatti and co-workers, there are several criteria for an effective molecular switch system. First, the switch should be an "ON-switch", meaning the switch should be able to be turned on and off based on the addition or removal of drug. Second, the drug and the molecular switch should be target specific and not interfere with endogenous metabolic pathways. Third, target gene expression should correlate with the dose of the ligand, which should rapidly reverse protein expression upon removal of ligand. Finally, the system should have low basal activity, be inactive in the absence of the ligand but strongly stimulated by ligand administration, hence high fold induction levels [18]. This paper characterizes a molecular switch system based on an orthogonal ligand/receptor pair that attempts to fulfill most of these requirements.

Nuclear receptors (NR) have the natural ability to bind ligands and regulate transcription. When a small molecule binds to a NR, a conformational change occurs in the receptor's structure allowing recruitment of the transcription machinery. This role in transcription makes NR crucial for the induction of gene expression and regulating a variety of cellular processes such as proliferation, differentiation, intracellular signalling, reproduction, and metabolism [19,20]. The modularity of nuclear receptors makes them attractive candidates for molecular switch systems; the ability for NR domains to function independently of each other allows them to be fused to other proteins for various protein engineering applications.

When engineering NR, the DNA binding domain (DBD) of these receptors can be engineered to recognize an artificial promoter containing multimeric-binding sites and a minimal promoter [1,2,21]. The ligand binding domain (LBD) can also be mutated to bind a synthetic small molecule that can reversibly regulate expression of genes. In addition to binding the synthetic ligand, NR can also be modified to eliminate their ability to bind their natural ligand. Several cases have shown the pairing of an unnatural or synthetic ligand with a mutant transcription factor [22]. In these systems, the small molecule binds to the mutant receptor and activates expression of a target transgene. These ligand/receptor pairs are ideal for molecular switch systems because of their selectivity and lack of interaction in other cellular pathways.

Previously, RXR was engineered to be regulated by LG335, a synthetic inactive analog of the compound LGD1069 (also known as bexarotene, trade name Targretin^®^) [23,24]. RXR belongs to the class of retinoid receptors [25], and plays important roles in cellular morphogenesis and differentiation [26,27]. Structurally, RXR contains a ligand binding domain (LBD) that recognizes various endogenous small lipophilic compounds such as 9-*cis*-retinoic acid (9cRA), and a DBD that recognizes a DNA sequence called RXR response elements (RE) [28]. When residues in the RXR LBD were mutated, one RXR variant Q275C, I310M, F313I (QCIMFI) had reverse ligand specificity, activating with LG335 but not with the wild type (wt) ligand, 9cRA [29]. This RXR variant can be further engineered to behave as a molecular switch and control gene expression in cell culture. This work focuses on the characterization of this molecular switch system in a two-component system and then this system is combined into a one-component system for stable expression in cell culture.

## Results

### Characterization of the two-component molecular switch system

**Figure 1:**
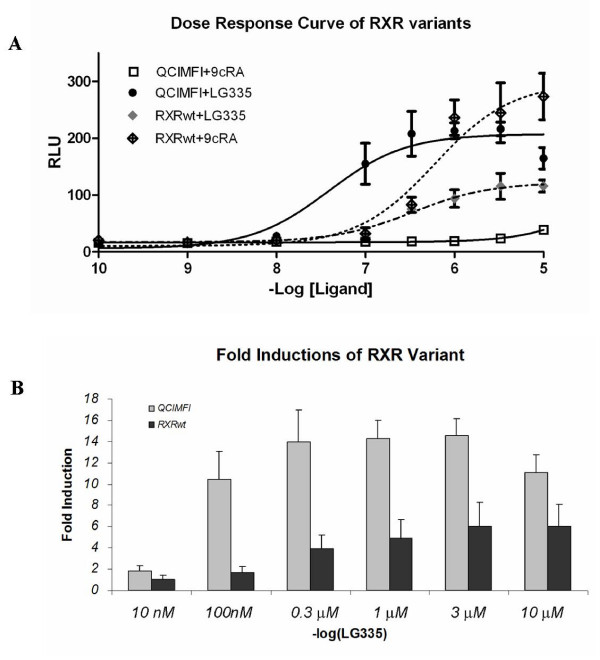
**Dose Response Curves for RXR Variants**. (A) Dose response curves for the activation of wild-type RXR DBD and LDB (RXRwt) and RXR variant Q275C;I310M;F313I with RXR DBD (QCIMFI) in response to 9cRA and LG335. Activity is measured in relative light units (RLU) derived from the measurement of luciferase activity and normalization against a *β*-galactosidase internal standard. Basal activity is around 20 RLUs. (B) Fold inductions of RXRwt and QCIMFI in the presence of various LG335 concentrations.

The molecular switch system was designed to contain an engineered NR, a promoter region, and a target transgene. Previously, Doyle *et al*. showed that a RXR variant consisting of three mutations in the LBD, Q275C, I310M, F313I (QCIMFI), and a unchanged DBD activated transcription in response to LG335 but not by the RXR natural ligand, 9cRA [29]. For determining the luciferase activation profile of this variant, a reporter plasmid containing the CRBPII response element controlling expression of the luciferase gene (pLuc_CRBPII) is used [28]. QCIMFI is activated in response to LG335 at concentrations as low as 100 nM (EC_50 _value is 38 nM) with a 14.5 ± 1.6-fold induction and no activation with 9cRA (Figure 1A). Conversely, RXRwt is activated by 1 μM 9cRA (EC_50 _value is 597 nM) with a 13.4 ± 4.2-fold induction and is activated by LG335 at the same concentration (EC_50 _value is 338 nM) but only at a fold induction of 6.0 ± 2.3. Figure 1B shows the fold induction at different concentrations of LG335 with QCIMFI and RXRwt. QCIMFI is activated at lower concentrations of LG335 and has much higher fold inductions than RXRwt. These results led to the development of a molecular switch using the QCIMFI variant and the ligand LG335.

As previously mentioned, one criterion for an effective molecular switch system is for the molecular switch to be target specific, without interference with endogenous pathways. To address this issue, the DBD of the receptor was switched from a RXR DBD to a Gal4 DBD. Gal4 is a yeast transcription factor that consists of two domains, a DNA binding domain and an activation domain. The DBD binds to four multiple repeats of a 17-mer DNA sequence called Gal4 RE [30]. This sequence, unique to yeast, provides specificity to a target promoter region containing Gal4 RE, and should not bind to endogenous mammalian DNA sequences. Thus, the fusion of the Gal4 DBD and the LBD of the RXR variant (GRQCIMFI) creates a new transcription factor involved in the molecular switch system addressed in this paper.

**Figure 2:**
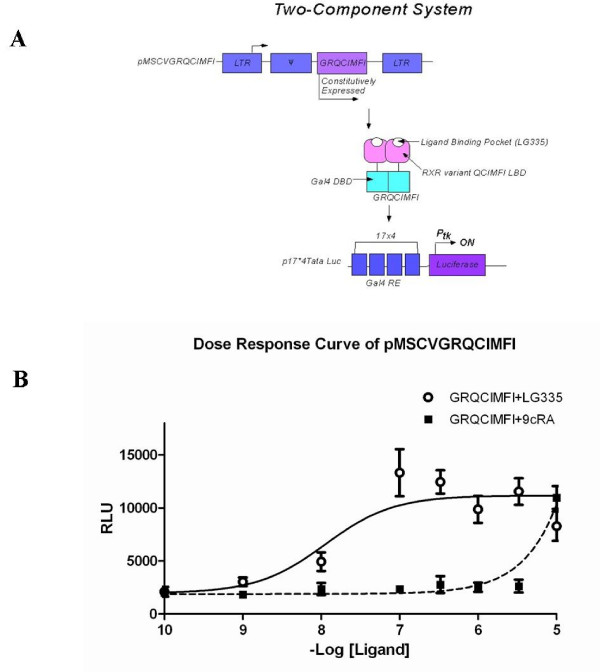
**Two-Component Molecular Switch System**. (A) Schematic diagram of a two-component molecular switch system. The engineered nuclear receptor GRQCIMFI (Gal4 DBD-RXR variant QCIMFI LBD) is constitutively expressed from plasmid pMSCVGRQCIMFI; and in the presence of ligand LG335, GRQCIMFI will bind to the Gal4 response elements (Gal4 RE). Gal4 RE is a 17-mer sequence that is repeated four times and is in front of the thymdine kinase promoter (P_*tk*_). The Gal4 RE as well as the target gene is in the plasmid p17*4TataLuc. When GRQCIMFI is bound to Gal4 RE the transcription machinery is recruited and the target gene, *Renilla *luciferase, is expressed. Therefore, the expression of the target gene is ligand-activated. (B) Luciferase assay of GRQCIMFI in the presence of LG335 (-◆-) or 9cRA (---■---). Basal activity is around 2000 RLUs.

In an effort to eventually introduce stable expression, GRQCIMFI was cloned into a retroviral expression vector, pMSCV, and the vector was renamed pMSCVGRQCIMFI. In this vector GRQCIMFI is constitutively expressed under the control of enhancers and a promoter in the long terminal repeat (LTR) region. Upon binding of LG335, GRQCIMFI can bind to a separate plasmid, p17*4TataLuc, containing four tandem Gal4 RE located upstream from a minimal thymidine kinase promoter (P_*tk*_), which induces expression of the *Renilla *luciferase gene (Figure 2A).

To assess the activation of our molecular switch system, pMSCVGRQCIMFI and p17*4TataLuc were cotransfected into HEK293T cells at a 1:2 molar ratio respectively, and tested with a range of ligand concentrations. As shown in Figure 2B, the two-component system induces expression of luciferase at 100 nM LG335 (EC_50 _value is 11 nM) leading to a 6.3 ± 1.7-fold induction ratio of luciferase activity, whereas activation with 9cRA only occurs at the highest concentration of ligand, 10 μM 9cRA (EC_50 _value is above 10 μM).

**Figure 3:**
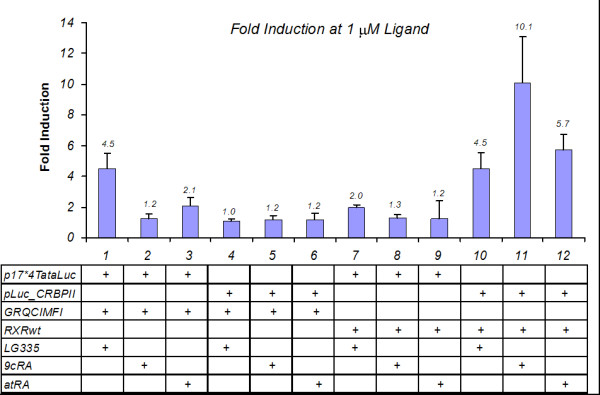
**GRQCIMFI Orthogonality**. Luciferase assays in the presence of 1 μM different ligands: LG335, 9cRA, or atRA. Constitutively expressed nuclear receptor, GRQCIMFI or RXRwt were cotransfected with p17*4TataLuc or pLuc_CRBPII. These plasmids have either Gal4 or RXR response elements respectively as well as the target gene, luciferase. Fold inductions for this assay are reported and normalized to wells with no ligand.

To further test the selectivity of GRQCIMFI to the Gal4 RE and LG335, GRQCIMFI was tested with an endogenous RE and compounds known to activate RXR. A combination of plasmids containing GRQCIMFI or RXRwt (pMSCVGRQCIMFI or pCMXRXR) along with reporter plasmid (p17*4TataLuc or pLuc_CRBPII) were cotransfected into HEK293T cells at a 1:2 molar ratio respectively with no ligand and with 1 μM ligand (LG335, 9cRA, and *all-trans *retinoic acid (atRA)). The plasmid pCMXRXR contains full length RXRwt under the control of a cytomegalovirus (CMV) promoter, and the pLuc_CRBPII plasmid contains RXR response elements controlling expression of *firefly *luciferase. As shown in Figure 3, the cotransfection of pMSCVGRQCIMFI and p17*4TataLuc results in a 4.5 ± 1.1-fold induction in the presence of 1 μM LG335, only a 1.2 ± 0.3-fold induction is observed with 9cRA, and a 2.1 ± 0.5-fold induction with atRA. As expected, activation does not occur when pMSCVGRQCIMFI is cotransfected with pLuc_CRBPII due to the fact that the Gal4 DBD does not bind to the RXR RE. Conversely, when pCMXRXR and pLuc_CRBPII plasmids are cotransfected, the highest activation occurs in the presence of the natural ligands, 9cRA and atRA, with fold inductions of 10.1 ± 3.0 and 5.7 ± 1.0, respectively. When pCMXRXR is cotransfected with p17*4TataLuc, minimal activation occurs since the RXR DBD does not recognize the Gal4 RE. The lack of activation of the molecular switch with endogenous RE and ligands shows that the engineered transcription factor has specificity to its target enhancer region and is orthogonal to the ligand, LG335.

### Ligand time course

**Figure 4:**
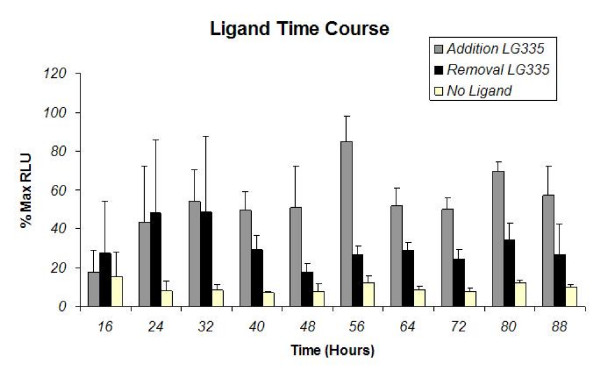
**Ligand Time Course**. HEK293T cells were transfected with pMSCVGRQCIMFI and p17*4TataLuc and 8 hours after transfection 100 nM of LG335 was added to cells. Cells were harvested every 8 hours for the ligand time course. Grey bars represent the addition of LG335 8 hours after transfection. The black bars represent the addition of LG335 8 hours after transfection and the removal of ligand 32 hours after. The white bars represent cells transfected with plasmid, but no ligand was added.

The time course of the ligand LG335 was assessed to determine the effect when ligand is continuously present in the cells 8 hours after transfection, as well as the removal of ligand 32 hours after transfection. Experimental results from several data sets were averaged (Figure 4), where HEK293T cells were cotransfected with pMSCVGRQCIMFI and p17*4TataLuc at a 1:2 molar ratio. All the cells received 100 nM LG335 eight hours after transfection; however, subsets of cells were washed with growth media 32 hours after transfection to remove LG335. As shown in Figure 4, when ligand is not removed from the medium, luciferase activity is detected within 24 hrs and activation increases 56 hours after transfection. A slight decrease in luciferase activity is observed after 56 hours, which could be due in part to the viability of the cells. However, when ligand is removed 32 hours after transfection, an immediate decrease in luciferase activity is observed at the next time point. These results show LG335 can induce transgene activation within 24 hours of adding ligand, and the ligand activation increases for about 56 hours after induction.

### Characterization of the one-component molecular switch system

**Figure 5:**
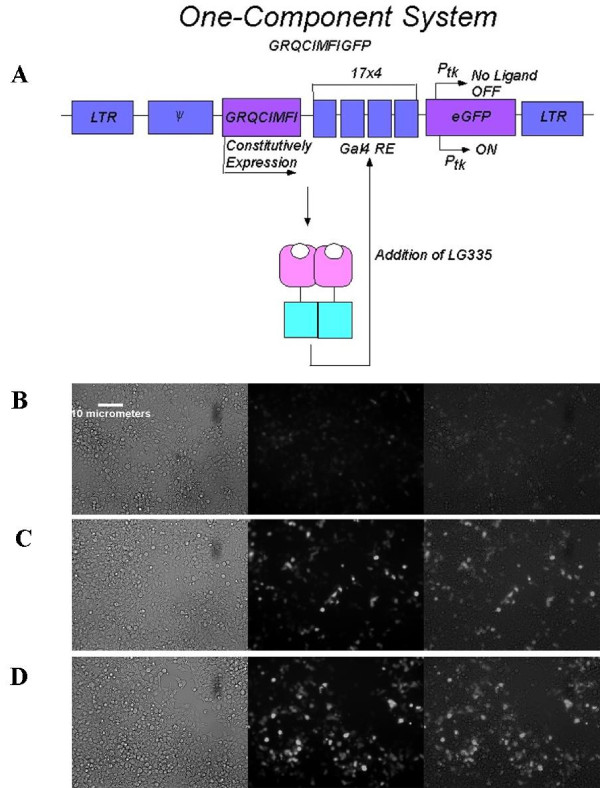
**One-Component System**. (A) Schematic diagram of one-component molecular switch system. All components of the molecular switch system were cloned into a pMSCV vector with eGFP as the target gene called GRQCIMFIGFP. (B, C, and D) HEK293T cells were transfected with GRQCIMFIGFP and (B) no ligand (C) 10 nM, or (D) 10 μM of LG335. Pictures were taken 40 hours after transfection and 32 hours after adding ligand.

As shown in the previous sections, the two-component system is capable of regulating gene expression; however, cotransfecting two plasmids is less desirable than transfecting a single plasmid. Any given cell needs both plasmids for the molecular switch to function, causing possible complications when administering this system in therapeutic applications. To make the system more versatile for stable expression in cell culture, all parts of the four kilobase sequence of the two-component system were cloned into the pMSCV vector, called GRQCIMFIGFP. In this vector, GRQCIMFI is constitutively expressed and in the presence of ligand induces expression of the enhanced green fluorescent protein (eGFP) (Figure 5A). The reporter gene was switched from luciferase to eGFP, since eGFP has the advantage of visualization of protein expression.

To evaluate the one-component system, GRQCIMFIGFP was transiently transfected into HEK293T cells with no ligand, 10 nM and 10 μM LG335. These results were compared to a control plasmid pMSCVIRESGFP, which constitutively expresses eGFP and contains an internal ribosomal entry site (IRES) prior to the eGFP DNA sequence (results not shown), to evaluate transfection efficiency. IRES is a DNA sequence that initiates translation of RNA by recruiting ribosomal subunits to a site on the RNA other than the 5' end [31,32]. Due to the continuous expression of the eGFP in the pMSCVIRESGFP plasmid, the protein expression levels are expected to be higher than that of our molecular switch. The transfection efficiency with the IRESGFP plasmid is approximately 60% (data not shown). The results in Figure 5B show that without ligand, basal GFP expression is observed where approximately 7% of the cells are dimly fluorescent. Upon the addition of 10 nM LG335 (Figure 5C) the intensity of the fluorescence increases and approximately 10% exhibit GFP expression. In the presence of 10 μM LG335 the expression of GFP is detected in about 30% of HEK293T cells (Figure 5D). In comparing the one-component to the two-component system, we find that both systems can be used as a reliable molecular switch. However, the one-component system may be more suitable because of the increased efficiency in cellular delivery.

### Characterizing integration and stable expression of the molecular switch in NIH3T3 cell line

After characterizing transient expression of the one-component system, the next step was to analyze stable expression of the molecular switch. One way to introduce stable expression of the molecular switch system is to infect cells using a retrovirus. Retroviral transductions allow the molecular switch's DNA to be integrated into the genome. Stable integration of the transgene allows for the testing of target gene expression over an extended period of time and after multiple cell passages.

**Figure 6:**
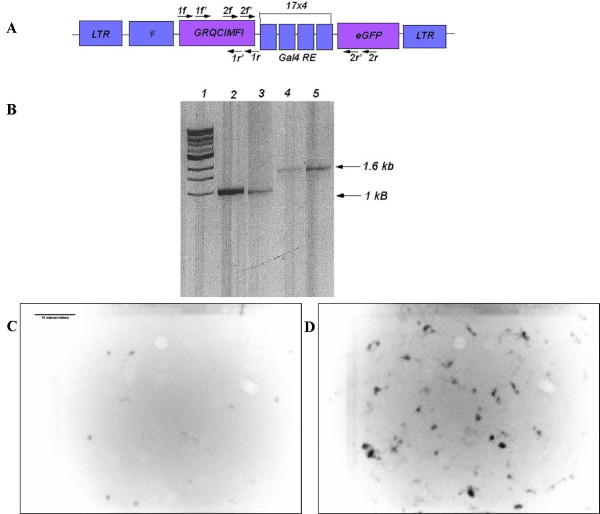
**Integration of GRQCIMFIGFP**. Genomic DNA was extracted from NIH3T3 cells transduced with GRQCIMFIGFP virus. PCR on genomic DNA shows that retroviral genes were integrated into the genome of cells. (A) The retroviral construct and the places where the different primers bind within the retrovirus construct. (B) An electrophoresis gel of the PCR product from genomic DNA (lanes 2 and 5) and plasmid DNA (lanes 3 and 4). Using primers 1f and 1r and then 1f' and 1r' to clone out a 1029 bp band (lanes 2 and 3). Primers 2f and 2r and then 2f' and 2r' were used to clone out a 1650 bp band (lanes 4 and 5). Lane is an l kb molecular weight marker. Figure (C and D) are pictures of NIH3T3 cells transduced with GRQCIMFIGFP with (C) no ligand and (D) 10 μM LG335.

To generate retrovirus, the ecotropic retroviral vector, GRQCIMFIGFP, was transiently transfected into the EcoPack 293 packaging cell line and infectious retroviral particles were collected and transduced into NIH3T3 cells. The multiplicity of infection used to infect cells was 0.44. These cells were then analyzed for integration of the virus into the cellular genome. To determine integration of the molecular switch sequence, a genomic extraction of NIH3T3 cells was collected and analyzed by nesting PCR. As shown in Figure 6A, PCR experiments were performed with primers that annealed to separate regions of the four kilobase one-component system. Primer sets "1" and "2" were used in PCRs with genomic DNA, and secondary PCRs were done with primer sets "1' " and "2' " to eliminate non-specific binding to genomic DNA sequences. As a positive control, these experiments were performed alongside plasmid DNA. Figure 6B shows the PCR fragments from the genomic DNA are the same size as predicted, suggesting that cellular integration occurs without transgene rearrangement. Genomic PCR fragments were also confirmed by sequencing. These results indicated that administrating this system through a retrovirus successfully integrated the molecular switch sequence into target cells.

To assess the regulation of the stable molecular switch system, NIH3T3 cells transduced with GRQCIMFIGFP were grown in media with no LG335 or 10 μM LG335 for 24 hours. These results show no GFP fluorescence is observed when no ligand is applied (Figure 6C), whereas 34% of the cells are fluorescent upon the addition of 10 μM LG335 (Figure 6D), further confirming this system as a useful tool for controlling gene expression.

## Discussion

One of the most widely used methods to control gene expression is molecular switch systems. These systems are composed of a transcription factor that interacts with a small molecule and an unnatural promoter [18], which produce relatively high activation levels. These systems are promising for research as well as clinical applications because they have been used for analyzing gene function in both cell culture as well as animal models [33,34].

As discussed previously, three successful ligand-dependent molecular switch systems have been used for various cellular studies; however, these systems contain certain limitations for *in vivo *applications. For example, the tetracycline-dependent system is composed of proteins from bacteria that may induce an immune response [12,13]. The GeneSwitch^® ^system uses RU486, a progesterone receptor (PR) antagonist and female contraceptive drug [35]; long term usage of this drug could lead to significant side effects, making this system difficult for animal studies [34]. The EcR-responsive system requires the heterodimerization of EcR with RXR. RXR is a reluctant dimer partner of EcR and over expression of RXR leads to potential interference in a plethora of metabolic pathways [36-38].

To address some of these disadvantages, a molecular switch system was developed using an orthogonal ligand/NR pair: LG335 and GRQCIMFI. This ligand/receptor pair is a sensitive switch showing activation of transgene at LG335 levels as low as 100 nM and activation was only observed with the wild-type ligand at very high concentrations. GRQCIMFI is constitutively expressed, and in the presence of ligand can activate expression of a target gene 6-fold. GRQCIMFI displays orthogonal behavior with LG335 and binds specific DNA sequences called Gal4 RE, exhibiting tight control over the target gene. Activation occurs only when pMSCVGRQCIMFI and p17*4TataLuc were cotransfected and 100 nM LG335 was added to the cells. No activation above basal activity occurred when pMSCVGRQCIMFI was transfected under different conditions. Wild type RXR shows high activation with the natural ligand, 9cRA; activation was also observed with LG335, but only at high concentrations. The ligand LG335 is a derivative of the FDA clinically approved drug bexarotene, but only bexarotene has biological relevance and can activate RXR [39,40].

As stated in the introduction, there are certain criteria for a molecular switch system. Some of these criteria include the turning on and off of transgene expression upon the addition of ligand. This system shows activation with two different reporters when LG335 was added; however, slight basal levels occur with both reporters when no ligand had been added. The ligand should be target specific and not interfere with endogenous pathways. We have shown that GRQCIMFI does not bind to RXR RE. Slight activation is observed with RXRwt but only at the highest concentration of ligand. It has also been shown that 10 μM 9cRA actives GRQCIMFI, but this concentration is much higher than cellular levels; therefore, it is expected that 9cRA will not activate this molecular switch. Lastly, the ligand should show rapid reversible transgene activation. This molecular switch system shows rapid induction, where activation levels are observed 16 hours after the addition of ligand. However, the removal of LG335 shows a slow and steady decrease in transgene expression. Possible improvements in this system can be achieved by adding a transactivation domain to increase activation levels [41], adding an insulator sequence to overcome promoter position effect [42], or adding an IRES sequence to increase the translation efficiency [43].

Initially, the molecular switch system was a two-component system with GRQCIMFI in one plasmid and the artificial promoter and target transgene in another plasmid. Then the system was combined into a one-component system containing all parts of the switch in one plasmid. While both systems were shown to regulate target gene expression proficiently, a one-component system provides versatility and efficiency by decreasing the amount of exogenous DNA required for the system to function [44]. The one-component system can be introduced virally into NIH3T3 cells and can stably express proteins. Integration of the molecular switch system into the genome is one way to introduce stable expression into a cellular environment while also passing the system's DNA material onto newly regenerated cells.

## Conclusions

Our data reveals a promising molecular switch system that drives controlled expression of two different reporter genes, luciferase and GFP, with high sensitivity and comparable induction ratios to other molecular switch systems. This system can be used in cell culture to assess gene expression of an array of target genes.

## Methods

### Ligands

9-*cis*-retinoic acid (MW 304.44 g/mol) and all-*trans*-retinoic acid (MW 300.44 g/mol) were purchased (ICN Biomedicals, USA). LG335 was synthesized in our lab [45,46].

### Plasmids

The pMSCVGRQCIMFI plasmid was constructed via PCR amplification of the Gal4 DBD and RXR LBD variant from the pGBDRXRQCIMFI plasmid using primers and *BglII *restriction sites. p17*4TataRluc was constructed from p17*4TataFLuc (a gift from Dr Sofia Tsai, Baylor College, Houston, TX) [47,48] by replacing the *firefly *luciferase with *Renilla *luciferase. The *Renilla *luciferase was cloned from pHRL (Clontech, USA) with *NotI *and *SacII *restriction sites. The internal standard plasmid pCMX-βGAL constitutively expresses β-galactosidase under control of the CMV promoter. The plasmids pCMXRXRwt and pCMXQCIMFI have been previously described [29]. The pLuc_CRPBII was made by site-directed mutagenesis from pLucMCS (Stratagene, USA). Site-directed primers were designed to incorporate a CRBPII response element in the multiple cloning site (MCS).

### Cell culture conditions

All cell types were maintained at 37°C in humidified air with 5% CO_2_. NIH3T3 (ATCC, USA) and HEK293T (ATCC, USA) cells were cultured in Dulbecco's Modified Eagle Medium (DMEM, Mediatech Inc, USA) supplemented with 10% calf bovine serum (CBS, Thermo Scientific, USA) and 1% penicillin/streptomycin (PS, VWR, USA).

### Mammalian luciferase assays

Transfections of HEK293T cells were performed in 48-well plates with Lipofectamine 2000 (Invitrogen, USA) as the cationic lipid as recommended by the manufacturer. Briefly, 20 ng of pMSCVGRQCIMFI expression plasmid, 40 ng of p17*4TataLuc reporter plasmid, and 40 ng of pCMX-βGAL expression plasmid (used as an internal standard) were mixed with 0.3 μL of lipofectamine 2000 in 40 μL of Opti-Mem (Invitrogen, USA) reduced serum media per transfected well. After 30-60 minutes an additional 160 μL of Opti-Mem was added and the 200 μL mixture was added to a well previously washed with 250 μL of Opti-Mem. After 8 hours of transfection the wells were aspirated and ligands diluted in growth media were added to the wells. Cells were harvested after 36-40 hours and assayed for luciferase and β-galactosidase activities. All data points represent the mean of triplicate experiments normalized against β-galactosidase activity. Error bars represent the standard deviation. All experiments were carried out in triplicate sets.

### Ligand time course

The transfection for this assay was done the same as stated above in the luciferase assay. However, cells were harvested every 8 hours for luciferase and β-galactosidase activity. Two experimental data sets were taken and each set was divided by the maximum RLUs and multiplied by 100 to receive the percent maximal RLUs. Then the average and standard deviation of both sets were calculated.

### Mammalian GFP analysis

Transfections of HEK293T cells were performed in 12-well plates with Lipofectamine 2000 cationic lipid as recommended by the manufacturer. Briefly, 1.6 μg of pMSCVGRQCIMFIGFP reporter plasmid was mixed with 4 μL of lipofectamine 2000 in 200 μL of Opti-Mem reduced serum media per transfected well. After 30-60 minutes an additional 1600 μL of Opti-Mem was added and the 2 mL mixture was added to a well previously washed with 2 mL of Opti-Mem. After 8 hours of transfection the wells were aspirated and ligands diluted in growth media were added to the wells. Images of transfected cells were taken using a 40× objective on a Zeiss LSM microscope. To obtain the percentage of fluorescent cells, the number of fluorescent cells counted was divided by the total number of cells counted multiplied 100. Images were processed on Adobe Photoshop to convert green fluorescence to gray-scale. All experiments were carried out in triplicate sets.

### Retrovirus

16 μg of pMSCVGRQCIMFIGFP was transiently transfected into EcoPack-293T cells (Clontech, USA) with 20 μL of lipofectamine 2000 and 6 mL of Opti-Mem. After eight hours, the media was changed to 7 mL of growth media. Collected viral particles in media on cells every 10-15 hours and filtered with a 0.45 μm syringe filter (Pall Corporation, USA). Transduction was done with 1 mL of media containing virus. Media was incubated with 80 μg/mL of chondroitin 6-sulfate sodium salt from shark cartilage (CSC, Sigma Aldrich, USA) for 10 minutes, and then with 80 μg/mL of polybrene (PB, Millipore Corporation, USA) for 10 minutes. Add media to 6-well plate of NIH3T3 cells with 8 μg/mL of polybrene. To obtain the percentage of fluorescent cells, the number of fluorescent cells counted was divided by the total number of cells counted multiplied by 100. Images of transduced cells were taken using a 40× objective on a Zeiss LSM microscope. To invert the images to gray scale, Adobe Photoshop was used. All experiments were carried out in duplicate sets.

### Genomic PCRs

Genomic DNA was collected using the DNeasy kits (Qiagen, USA). To clone the 1029 bp DNA sequence from genomic DNA, a primary PCR was performed using the following primers: 1f, CCT TGA CAT GAT TTT GAA AAT GG; 1r, GCC GCC TAA GTC ATT TGG TG. Then a secondary PCR was performed with the following primers: 1f', ATT CTT TAC AGG ATA TAA AAG CAT TGT TAA CAG GAT; 1r', CGC CTC CAG CAT CTC CAT AAG G. To clone out the 1650 bp DNA sequence, a primary PCR was done with the following primers: 2f, GAG GTG GAG TCG ACC AGC AG; 2r, TTA CTT GTA CAG CTC GTC CAT GC. A secondary PCR was done with the following primers: 2f', CGC CAA CGA GGA CAT GCC G; 2r', CGA GAG TGA TCC CGG CGG C. *Pfu *polymerase (Stratagene, USA) was used. The PCR fragments were analyzed on a 1.2% agarose gel.

## Abbreviations

NR: nuclear receptors; RXR: retinoid × receptor; DBD: DNA binding domain; LBD: ligand binding domain; RE: response element; 9cRA: 9-*cis*-retinoic acid; atRA: *all-trans *retinoic acid; eGFP: enhanced green fluorescent protein; PR: progesterone receptor; RU486: mifepristone; Tet: tetracycline; dox: doxycycline; EcR: ecdysone receptor; wt: wild type; CMV: cytomegalovirus.

## Authors' contributions

JLT conceived and designed the study, carried out all the collection, analysis and interpretation of data, and drafted the manuscript. PR contributed to the design of the study and the technical direction of the experiments. THS, DFD, and BA contributed to the conception and design of the study, and the preparation of the manuscript. All authors read and approved the final manuscript.

## References

[B1] Pollock R Clackson T Dimerizer-regulated gene expression Curr Opin Biotechnol 2002 13 5 459 467 10.1016/S0958-1669(02)00373-7 12459338

[B2] Clackson T Controlling mammalian gene expression with small molecules Curr Opin Chem Biol 1997 1 2 210 218 10.1016/S1367-5931(97)80012-9 9667854

[B3] Wang YL Omalley BW Tsai SY A regulatory system for gene-transfer Proc Natl Acad Sci USA 1994 91 17 8180 8184 10.1073/pnas.91.17.8180 8058776 PMC44569

[B4] Gossen M Bujard H Tight control of gene-expression in mammalian-cells by tetracycline-responsive promoters Proc Natl Acad Sci USA 1992 89 12 5547 5551 10.1073/pnas.89.12.5547 1319065 PMC49329

[B5] No D Yao TP Evans RM Ecdysone-inducible gene expression in mammalian cells and transgenic mice Proc Natl Acad Sci USA 1996 93 8 3346 3351 10.1073/pnas.93.8.3346 8622939 PMC39610

[B6] Vegeto E Allan GF Schrader WT Tsai MJ McDonnell DP Omalley BW The mechanism of RU486 antagonism is dependent on the conformation of the carboxy-terminal tail of the human progesterone-receptor Cell 1992 69 4 703 713 10.1016/0092-8674(92)90234-4 1586949

[B7] Wang Y Xu J Pierson T Omalley BW Tsai SY Positive and negative regulation of gene expression in eukaryotic cells with an inducible transcriptional regulator Gene Therapy 1997 4 5 432 441 10.1038/sj.gt.3300402 9274720

[B8] Burcin MM Schiedner G Kochanek S Tsai SY O'Malley BW Adenovirus-mediated regulable target gene expression in vivo Proc Natl Acad Sci USA 1999 96 2 355 360 10.1073/pnas.96.2.355 9892637 PMC15140

[B9] Nordstrom JL The antiprogestin-dependent GeneSwitch (R) system for regulated gene therapy 2003 2003: Elsevier Science Inc 1085 1094 14668002 10.1016/j.steroids.2003.07.008

[B10] Salucci V Scarito A Aurisicchio L Lamartina S Nicolaus G Giampaoli S Gonzalez-Paz O Toniatti C Bujard H Hillen W Ciliberto G Palombo F Tight control of gene expression by a helper-dependent adenovirus vector carrying the rtTA2(s)-M2 tetracycline transactivator and repressor system Gene Therapy 2002 9 21 1415 1421 10.1038/sj.gt.3301813 12378403

[B11] Zhu Z Zheng T Lee CG Homer RJ Elias JA Tetracycline-controlled transcriptional regulation systems: advances and application in transgenic animal modeling Semin Cell Dev Biol 2002 13 2 121 128 10.1016/S1084-9521(02)00018-6 12127145

[B12] Favre D Blouin V Provost N Spisek R Porrot F Bohl D Marme F Cherel Y Salvetti A Hurtrel B Heard JM Riviere Y Moullier P Lack of an immune response against the tetracycline-dependent transactivator correlates with long-term doxycycline-regulated transgene expression in nonhuman primates after intramuscular injection of recombinant adeno-associated virus J Virol 2002 76 22 11605 11611 10.1128/JVI.76.22.11605-11611.2002 12388721 PMC136781

[B13] Latta-Mahieu M Rolland M Caillet C Wang MP Kennel P Mahfouz I Loquet I Dedieu JF Mahfoudi A Trannoy E Thuillier V Gene transfer of a chimeric trans-activator is immunogenic and results in short-lived transgene expression Hum Gene Ther 2002 13 13 1611 1620 10.1089/10430340260201707 12228016

[B14] No D Yao TP Evans RM Ecdysone-inducible gene expression in mammalian cells and transgenic mice Proc Natl Acad Sci USA 1996 93 8 3346 3351 10.1073/pnas.93.8.3346 8622939 PMC39610

[B15] Karns LR Kisielewski A Gulding KM Seraj JM Theodorescu D Manipulation of gene expression by an ecdysone-inducible gene switch in tumor xenografts BMC Biotechnol 2001 1 11 10.1186/1472-6750-1-11 11782290 PMC64497

[B16] Palli SR Kapitskaya MZ Kumar MB Cress DE Improved ecdysone receptor-based inducible gene regulation system Eur J Biochem 2003 270 6 1308 1315 10.1046/j.1432-1033.2003.03501.x 12631289

[B17] Subbarayan V Mark M Messadeq N Rustin P Chambon P Kastner P RXRalpha overexpression in cardiomyocytes causes dilated cardiomyopathy but fails to rescue myocardial hypoplasia in RXRalpha-null fetuses J Clin Invest 2000 105 3 387 394 10.1172/JCI8150 10675365 PMC377445

[B18] Toniatti C Bujard H Cortese R Ciliberto G Gene therapy progress and prospects: transcription regulatory systems Gene Therapy 2004 11 8 649 657 10.1038/sj.gt.3302251 14985790

[B19] Germain P Staels B Dacquet C Spedding M Laudet V Overview of nomenclature of nuclear receptors Pharmacol Rev 2006 58 4 685 704 10.1124/pr.58.4.2 17132848

[B20] Sonoda J Pei LM Evans RM Nuclear receptors: Decoding metabolic disease FEBS Lett 2008 582 1 2 9 10.1016/j.febslet.2007.11.016 18023286 PMC2254310

[B21] Pollock R Giel M Linher K Clackson T Regulation of endogenous gene expression with a small-molecule dimerizer Nat Biotechnol 2002 20 7 729 733 10.1038/nbt0702-729 12089560

[B22] Bishop A Buzko O Heyeck-Dumas S Jung I Kraybill B Liu Y Shah K Ulrich S Witucki L Yang F Zhang C Shokat KM Unnatural ligands for engineered proteins: New tools for chemical genetics Annu Rev Biophys Biomolec Struct 2000 29 577 606 10.1146/annurev.biophys.29.1.577 10940260

[B23] Boehm MF McClurg MR Pathirana C Mangelsdorf D White SK Hebert J Winn D Goldman ME Heyman RA Synthesis of high specific activity [3H]-9-cis-retinoic acid and its application for identifying retinoids with unusual binding properties J Med Chem 1994 37 3 408 414 10.1021/jm00029a013 8308867

[B24] Boehm MF Zhang L Badea BA White SK Mais DE Berger E Suto CM Goldman ME Heyman RA Synthesis and structure-activity relationships of novel retinoid × receptor-selective retinoids J Med Chem 1994 37 18 2930 2941 10.1021/jm00044a014 8071941

[B25] Mangelsdorf DJ Evans RM The RXR heterodimers and orphan receptors Cell 1995 83 6 841 850 10.1016/0092-8674(95)90200-7 8521508

[B26] Mangelsdorf DJ Ong ES Dyck JA Evans RM Nuclear receptor that identifies a novel retinoic acid response pathway Nature 1990 345 6272 224 229 10.1038/345224a0 2159111

[B27] Shulman AI Mangelsdorf DJ Mechanisms of disease: Retinoid × receptor heterodimers in the metabolic syndrome N Engl J Med 2005 353 6 604 615 10.1056/NEJMra043590 16093469

[B28] Mangelsdorf DJ Umesono K Kliewer SA Borgmeyer U Ong ES Evans RM A direct repeat in the cellular retinol-binding protein type-II gene confers differential regulation by RXR and RAR Cell 1991 66 3 555 561 10.1016/0092-8674(81)90018-0 1651173

[B29] Doyle DF Braasch DA Jackson LK Weiss HE Boehm MF Mangelsdorf DJ Corey DR Engineering orthogonal ligand-receptor pairs from "near drugs" Journal of the American Chemical Society 2001 123 46 11367 11371 10.1021/ja0164632 11707111

[B30] Lohr D Venkov P Zlatanova J Transcriptional regulation in the yeast gal gene family - A complex genetic network Faseb J 1995 9 9 777 787 7601342 10.1096/fasebj.9.9.7601342

[B31] Kieft JS Viral IRES RNA structures and ribosome interactions Trends BiochemSci 2008 33 6 274 283 10.1016/j.tibs.2008.04.007 PMC2706518 18468443

[B32] Pfingsten JS Kieft JS RNA structure-based ribosome recruitment: Lessons from the Dicistroviridae intergenic region IRESes RNA-Publ RNA Soc 2008 14 7 1255 1263 10.1261/rna.987808 PMC2441983 18515544

[B33] Mulligan RC The basic science of gene therapy Science 1993 260 5110 926 932 10.1126/science.8493530 8493530

[B34] Goverdhana S Puntel M Xiong W Zirger JM Barcia C Curtin JF Soffer EB Mondkar S King GD Hu J Sciascia SA Candolfi M Greengold DS Lowenstein PR Castro MG Regulatable gene expression systems for gene therapy applications: Progress and future challenges Mol Ther 2005 12 2 189 211 10.1016/j.ymthe.2005.03.022 15946903 PMC2676204

[B35] Sarkar NN Mifepristone: bioavailability, pharmacokinetics and use-effectiveness Eur J Obstet Gynecol Reprod Biol 2002 101 2 113 120 10.1016/S0301-2115(01)00522-X 11858883

[B36] Thomas HE Stunnenberg HG Stewart AF Heterodimerization of the drosophila ecdysone receptor with retinoid × receptor and ultraspirale Nature 1993 362 6419 471 475 10.1038/362471a0 8385270

[B37] Yao TP Forman BM Jiang ZY Cherbas L Chen JD McKeown M Cherbas P Evans RM Functional ecdysone receptor is the product of ECR and ultraspiracle genes Nature 1993 366 6454 476 479 10.1038/366476a0 8247157

[B38] Yao TP Segraves WA Oro AE McKeown M Evans RM Drosophila ultraspiracle modulates ecdysone receptor function via heterodimer formation Cell 1992 71 1 63 72 10.1016/0092-8674(92)90266-F 1327536

[B39] Boehm MF Zhang L Badea BA White SK Mais DE Berger E Suto CM Goldman ME Heyman RA Synthesis and structure activity relationships of novel retinoid × receptor selective retinoids J Med Chem 1994 37 18 2930 2941 10.1021/jm00044a014 8071941

[B40] Boehm MF Zhang L Zhi L McClurg MR Berger E Wagoner M Mais DE Suto CM Davies PJA Heyman RA Nadzan AM Design and synthesis of potent retinoid × receptor selective ligands that induce apoptosis in leukemia-cells J Med Chem 1995 38 16 3146 3155 10.1021/jm00016a018 7636877

[B41] Ingles CJ Shales M Cress WD Triezenberg SJ Greenblatt J Reduced binding of TFIID to transcriptionally compromised mutants of VP16 Nature 1991 351 6327 588 590 10.1038/351588a0 1646402

[B42] Osti D Marras E Ceriani I Grassini G Rubino T Vigano D Parolaro D Perletti G Comparative analysis of molecular strategies attenuating positional effects in lentiviral vectors carrying multiple genes J Virol Methods 2006 136 1-2 93 101 10.1016/j.jviromet.2006.04.003 16690138

[B43] Szulc J Wiznerowicz M Sauvain MO Trono D Aebischer P A versatile tool for conditional gene expression and knockdown Nat Methods 2006 3 2 109 116 10.1038/nmeth846 16432520

[B44] Lattime EC Retroviral Vector Design for Cancer Gene Therapy Gene Therapy of Cancer 2002 2 San Diego: Academic Press 3 23

[B45] Schwimmer LJ Rohatgi P Azizi B Seley KL Doyle DF Creation and discovery of ligand-receptor pairs for transcriptional control with small molecules Proc Natl Acad Sci USA 2004 101 41 14707 14712 10.1073/pnas.0400884101 15456909 PMC522017

[B46] Schwimmer LJ Engineering ligand-receptor pairs for small molecule control of transcription 2005 Atlanta: Georgia Institute of Technology

[B47] Wang Y O'Malley BW Jr Tsai SY O'Malley BW A regulatory system for use in gene transfer Proc Natl Acad Sci USA 1994 91 17 8180 8184 10.1073/pnas.91.17.8180 8058776 PMC44569

[B48] Wang Y Xu J Pierson T O'Malley BW Tsai SY Positive and negative regulation of gene expression in eukaryotic cells with an inducible transcriptional regulator Gene Ther 1997 4 5 432 441 10.1038/sj.gt.3300402 9274720

